# Study of level of the precautionary measures taken by parents and the impact of COVID 2019 on children daily life during curfew

**DOI:** 10.1016/j.amsu.2022.103969

**Published:** 2022-08-02

**Authors:** Yusr Abdullah Alsadan, Jumana Safwan Gassem, Jumana Mazen Dera, Yara Mohamed Elbatanony, Nagham Mohamed El Beblawy, Ibrahim Mansour

**Affiliations:** a East Jeddah Hospital, Jeddah, Saudi Arabia; b Ibn Sina College, Jeddah, Saudi Arabia; c Aljadaani Hospital, Jeddah, Saudi Arabia

**Keywords:** Precautionary measures, Parents, The impact of covid 2019, Children, Daily life

## Abstract

**Background:**

COVID-19 has caused many changes in all communities world-wildly, at many levels, leaving us to reset our priorities and needs. All that for the sake of surviving this pandemic. But children played the least important role in these rearrangements, or at least this how the community handled it.

**Aim of the study:**

To study the precautionary measures that were taken by the parents on their children in the kingdom of Saudi Arabia during curfew. Study the impact of COVID on children's healthcare, diet, and daily routines.

**Methods:**

A community-based cross-sectional study was conducted in different regions of Saudi Arabia from the period of 1st July 2020 to 30th January 2021. Data were compiled and analyzed using a statistical package for the social sciences (SPSS, version 16) and results were analyzed with frequencies and Chi-squared test as appropriate. P-value was considered significant if P < 0.05.

**Results:**

The study included 532 participants, 69.7% of participated parents were mothers (females) and 30.3% were fathers (males). 83.5% of mothers were highly educated. 41% didn't observe any change in their children's activity, the little less 37% agreed that it decreased their activity. 64.5% of the parents believed that herbal meds and supplements boost their children's immunity. 27.6% were not sure. The rest 7.9% didn't believe in their effect. 39% of the parents who had children in the age of vaccination, had to delay it, while 60% stuck to the schedule.

**Conclusion:**

People sticking to precautionary measures were more relatable to what they believed not what they were forced to do the increased number of cases at the beginning of the partial curfew proves that. Even though social gathering had its financial penalty the responses were greater with hand washing.

## Highlights

• Study the impact of COVID on children's healthcare, diet, and daily routines.
• The majority 41% didn't observe any change in their children's activity.
• Children can be the secret host of COVID through witch is spreads fast without anyone knowing since they appear to present with less severe to no symptoms at all.
• People sticking to precautionary measures were more relatable to what they believed not what they were forced to do.
• Isolated from the big scene should be built to establish a continuity of the primary care of children, minimizing the collateral damage in this disaster.

## 1 Introduction

COVID is a novel coronavirus whose first case was reported in 2019 December in Wuhan city [1] in China. Since then, the virus showed a rapid spread in a short period resulting in nearly 37,000,000 positive cases causing more than 1,000,000 deaths worldwide up until now. On 30 Jan 2020, The Director-General declared the novel coronavirus outbreak a public health emergency of international concern (PHEIC), WHO's the highest level of alarm. Advising all the countries to take their measures to fight the spread of the virus. [[2], [3], [4], [5]]. COVID-19 has caused many changes in all communities worldwide, at many levels, leaving us to reset our priorities and needs. All that for the sake of surviving this pandemic [6]. But children played the least important role in these rearrangements, or at least this how the community handled it. Our research tries to put all the main concepts people have that are related to children during the outbreak. And if they were any misleading ones or even malpractices. On the 2nd of March 2020, the first case of COVID-19 was reported by the Saudi authorities. After that, all sports competitions would be held behind closed doors as of the 7th of March 2020 that was the beginning of the restriction the government applied on any gathering, starting in public places to the beginning f the curfew. That left people with one entertainment way which is the home social gathering. It was enough to cause an increase in the incidence of daily cases since then [7].From this point, we understand that the level of awareness and the commitment to follow the precautionary measures is as important as a strict system itself.

## 2 Aim of the study

To study the precautionary measures that were taken by the parents on their children in the kingdom of Saudi Arabia. Study the impact of COVID on children's healthcare, diet, and daily routines. The study also aims to compare between the Saudi Arabia regions on how strict they follow the precautions and to assess the impact of COVID-19 outbreak on the healthcare of the children at the level of each household and daily lifestyle of children.

## 3 Methodology

**Study design and duration**: Cross-sectional study was conducted during the period 1st July 2020 to 30th January 2021.

**Study area**: The study was conducted in different regions in Saudi Arabia.

**Study population**: Saudi parents living in Saudi Arabia during the COVID-19 outbreak.

**Sample size**: The minimum sample size for this study has been decided according to Swinscow, as follows:

$$n=\frac{Z^{2}\text{xPxQ}}{D^{2}}$$

where:

n minimum sample size
Z The z-value for the selected level of confidence (1- <) = 1.96.
P An estimated prevalence of having a positive attitude.
Q (1–0.50) = 50%, i.e., 0.50
D The maximum acceptable error = 0.05.

So, the calculated minimum sample size was:

n (minimum) = (1.96)2 X 0.50 X 0.50 = 384 (0.05) 2.

532 participants were included in the study from 3 different geographical regions in the kingdom.

**The Sampling Technique**: Random sampling technique was adopted to select the sample size.

**Data Collection Tool**: A self-administered online disseminated questionnaire was used for data collection. It is composed of two main sections. Section 1 includes socio-demographic characteristics of the parents (child sex, mother education, and residency). The second section asks about physical activity, food, vaccination delay, supplements benefit, susceptibility, symptoms, protective precautions to children, and supplements. Permission to utilize the questionnaire was asked from the two main authors through email.

**Data Collection Technique**: The researchers distributed the questionnaire online as the questionnaire will be distributed online on social media sites (WhatsApp- Facebook- Twitter) to be filled out personally. The questionnaire had a brief introduction explaining the nature of the research and confidentiality of the information that is given to participants.

**Data Management and Analysis Plan**: All data were analyzed using SPSS 23 with using appropriate statistical methods for description and analysis. A P-value less than 0.05 was considered for statistical significance.

**Statistical analysis**: Data were entered and analyzed using Statistical Package for the Social Sciences (SPSS) version 17. Descriptive statistics were displayed as frequencies and percentages for categorical variables. Univariate analysis was performed to compare between each region, with the outcome, on the one hand, this was performed using Chi-squared test.

## 4 Results

Our study included 532 participants, 69.7% of participated parents were mothers (females) and 30.3% were fathers (males). 83.5% of mothers were highly educated. Regarding residency, 54.1% of our sample were from the western region, 21.1% from the eastern region, 2.1% from the southern region, 0.8% from the northern region, and 22.0% from the central region. The majority 41% didn't observe any change in their children's activity, the little less 37% agreed that it decreased their activity. The least said that children seemed more active. 44%–95% of the respondents ate from what they cooked ranging from all to most of the weekdays, less than 5% stuck with other sources. 64.5% of the parents believed that herbal meds and supplements boost their children's immunity. 27.6% were not sure. The rest 7.9% didn't believe in their effect. 39% of the parents who had children in the age of vaccination, had to delay it, while 60% stuck to the schedule. The severity of the symptoms, if it hits children comparing to the adults, was reported as 67.7% had milder symptoms and 32.3% thought no the presented with the same picture as adults (as showen in Table 1)

**Table 1:** Table 1: Sociodemographic characteristics, precautionary measures and impact of covid 2019 on children daily.

	.	Frequency	Percent
Parent Gender	Father	161	30.3
	Mother	371	69.7
	Total	532	100.0
Mother Education	Didn't finish school	9	1.7
	High school graduate	79	14.8
	College	444	83.5
Residency	West	288	54.1
	Eastern	112	21.1
	Southern	11	2.1
	Central	117	22.0
Children	One	102	19.2
	Two to three	189	35.5
	more than three	241	45.3
Physical Activity	Increased	112	21.1
	Decreased	197	37.0
	No change	223	41.9
Food	always homemade food	237	51.7
	Most of the time homemade food	18	3.4
	Rarely homemade food	2	.4
Vaccination delay	Yes	74	13.9
	No	114	21.4
	No child to be vaccinated	344	64.7
Supplements benefit	Yes	343	64.5
	No	42	7.9
	Maybe	147	27.6
Symptoms	Yes	360	67.7
	No	172	32.3
Protective precautions for children	Hand wash	452	85.0
	No kid goes for groceries	366	68.8
	Face mask	423	79.5
	No kid receives a delivery	327	61.5
	Didn't have visitors	326	61.3
	Going out for important stuff	371	69.7
Supplements	Nothing	85	16.0
	Honey	347	65.2
	vitamins	208	39.1
	Honey, Black seed, vitamins	184	34.6
	Honey, Black seed	67	12.6
	Honey, vitamins	55	10.4
	Others	28	5.3

## 5 Discusion

The huge numbers of cases and deaths COVID caused had all our attention, but simple things like diet and physical activity that can help with fighting the pandemic as a whole are left aside. A study done showed that the physical activity of people got reduced by 25% [7] Among children and young people, there is compelling evidence suggesting that physical activity is important for health and well-being. Physical activity might improve not only cardiorespiratory and muscular fitness, cardiometabolic health, bone health, weight status, and cognition, but also reduce the risk of depression. The current physical activity recommendations suggest that children and young people aged 6–17 years should engage in 60 min/d or more of moderate-to-vigorous physical activity, of which vigorous physical activity should be included at least three days per week [8] Diet and supplementation play a major role in human immunity and it has an association in modulating the immune responses COVID symptomatology [9] That also got affected people who had more frequent carbohydrate meals. mean weight was significantly higher during the curfew than before the curfew([9]). On March 23, 2020, SPA – An official source in the Ministry of Interior has stated that based on the order of the Custodian of the Two Holy Mosques King Salman bin Abdul-Aziz Al Saud to enforcing a curfew to limit the spread of the Novel Coronavirus. This lasted not less than two months [10]. A study found that social distancing caused the case growth rate to reduce the total number of COVID-19 cases by approximately 1,600 reported cases at 7 days and the following days [5]. Plus the variation that the chart showed along the timeline of the curfew of KSA [11], had us put people behavior and actions into consideration when dealing with outbreaks in general. And with that disturbing the rhythm of daily life routine of everyone, we wanted to know what change COVID made on the families that live in KSA, and what measure did the parents take to protect their children. Starting from the physical activity of the children we asked the parents whether their children's activity got affected. Regardless of the scientific accuracy of the measures taken by the parents during the pandemic, we asked them withier they believed in their effect and if they practiced any. And those were the supplements and food that were given to enhance the children's immunity. Moving to the precautions the Ministry of Health set to follow, we wanted to see how strict each parent was in keeping up with them, and we found that a good percentage of responses claimed that they followed most of the precautions. They let every member of the house including children wash their hands that got the highest percentage 85% in the other hand the responses with the people who didn't have visitors at home or didn't let any of the children meet outside visitors were the least 61%. The primary immunization of children in KSA starts from birth till preschool age. It proved it is efficacy in eradicating some diseases and weakening others [12]. Far from here, It has been reported that the number of MMR (measles, mumps, and rubella) vaccines delivered in England dropped by 20% during the first three weeks of the lockdown,8 and smaller falls were reported in infant vaccines in Scotland [13]. Children can be the secret host of COVID through witch is spreads fast without anyone knowing since they appear to present with less severe to no symptoms at all [14,15]. Regardless of the absence of any study about the children's susceptibility to COVID, some parents believe that their children are more protected from COVID than the adults, and so we asked them if they believe in that. 45.7% yes they answered children were more protected. 54% said no they were as susceptible as adults The clinical presentation of covid-19 patients varies according to how each immune system will respond to it. The theory behind this is poorly understood although some research studies revealed major immunological parameter differences between individuals who tested positive [16], but the whole image is yet to be discovered. What we know so far are some theories based on many immunological studies that concluded that the age and the comorbidities of the patients are one of the main important players in the morbidity and mortality of the disease, but with no clear-cut lines [17,18].

## 6 Conclusion

People sticking to precautionary measures were more relatable to what they believed not what they were forced to do, the increased number of cases at the beginning of the partial curfew proves that. Even though social gathering had its financial penalty the responses were greater with hand washing. Since they believed that their children are at less risk. Of dying or danger when getting COVID they did not mind those meeting people during that. Last but not least as for the vaccination being sabotaged by this even an organized health care system, isolated from the big scene should be built to establish a continuity of the primary care of children, minimizing the collateral damage in this disaster.

## Please state any conflicts of interest

N/A.

## Please state any sources of funding for your research

N/A.

## Ethical approval

Institutional ethics committee Ibn Sina national college for medical studies. Jeddah. KSA. Title of the protocol: STUDY OF LEVEL OF THE PRECAUTIONS TAKEN BY PARENTS ON CHILDREN DURING COVID-19 PANDIMIC. Protocol Identification: 002SRC02082020.

## Consent

N/A.

## Author contribution

Dr. Yusr alsadan »>study concept or design, writing the paper The rest of the Authors»» data collection, data analysis or interpretation, writing the paper.

## Registration of research studies

N/A.

Name of the registry:

1. Unique Identifying number or registration ID:

2. Hyperlink to your specific registration (must be publicly accessible and will be checked):

## Guarantor

Dr.Yusr Abdullah Saleh ALsadan.
