# Supplementary material for: Study of level of the precautionary measures taken by parents and the impact of COVID 2019 on children daily life during curfew
Source: Ann Med Surg (Lond). 2022 Aug 2;80:103969. doi: 10.1016/j.amsu.2022.103969 (PMC9345652; doi:10.1016/j.amsu.2022.103969)
Supplement: Multimedia component 1 [file mmc1.docx]

| **The STROCSS 2021 Guideline** |
| --- |
| **Item description** |
| **TITLE** |
| **Title**  ***STUDY OF LEVEL OF THE PRECAUTIONARY MEASURES TAKEN BY PARENTS AND THE IMPACT OF COVID 2019 ON CHILDREN DAILY LIFE DURING CURFEW** |
| **ABSTRACT** |
| **Background:** COVID-19 has caused many changes in all communities world- wildly, at many levels, leaving us to reset our priorities and needs. All that for the sake of surviving this pandemic. But children played the least important role in these rearrangements, or at least this how the community handled it.  **Aim of the study:** To study the precautionary measures that were taken by the parents on their children in the kingdom of Saudi Arabia during curfew . Study the impact of COVID on children's healthcare, diet, and daily routines. |
| **Methods** -A community-based cross-sectional study was conducted in different regions of Saudi Arabia from the period of 1st July 2020 to 30th January 2021. Data were compiled and analyzed using a statistical package for the social sciences (SPSS, version 16) and results were analyzed with frequencies and Chi-squared test as appropriate. P-value was considered significant if P <0.05. |
| **Results** -The study included 532 participants, 69.7% of participated parents were mothers (females) and 30.3% were fathers (males). 83.5% of mothers were highly educated. 41% didn’t observe any change in their children’s activity, the little less 37% agreed that it decreased their activity. 64.5% of the parents believed that herbal meds and supplements boost their children's immunity. 27.6% were not sure. The rest 7.9% didn’t believe in their effect. 39% of the parents who had children in the age of vaccination, had to delay it, while 60 % stuck to the schedule. |
| **Conclusion** People sticking to precautionary measures were more relatable to what they believed not what they were forced to do the increased number of cases at the beginning of the |
| **INTRODUCTION** |
| **I**COVID is a novel coronavirus whose first case was reported in 2019 December in Wuhan city (1) in China. Since then, the virus showed a rapid spread in a short period resulting in nearly 37,000,000 positive cases causing more than 1,000,000 deaths worldwide up until now (2-4). On 30 Jan 2020, The Director-General declared the novel coronavirus outbreak a public health emergency of international concern (PHEIC), WHO's the highest level of alarm. Advising all the countries to take their measures to fight the spread of the virus.  COVID-19 has caused many changes in all communities worldwide, at many levels, leaving us to reset our priorities and needs. All that for the sake of surviving this pandemic. But children played the least important role in these rearrangements, or at least this how the community handled it. Our research tries to put all the main concepts people have that are related to children during the outbreak. And if they were any misleading ones or even malpractices.  On the 2nd of March 2020, the first case of COVID-19 was reported by the Saudi authorities. After that, all sports competitions would be held behind closed doors as of the 7th of March 2020 that was the beginning of the restriction the government applied on any gathering, starting in public places to the beginning f the curfew. That left people with one entertainment way which is the home social gathering. It was enough to cause an increase in the incidence of daily cases since then (5). From this point, we understand that the level of awareness and the commitment to follow the precautionary measures is as important as a strict system itself. |
| **METHODS** |
| **Registration : research registry number : 7528**  Protocol Identification: 002SRC02082020 |
| **Ethical approval** |

| **Protocol**  Institutional ethics committee  Ibn Sina national college for medical studies.  Jeddah. KSA.  Title of the protocol : STUDY OF LEVEL OF THE PRECAUTIONS TAKEN BY PARENTS ON CHILDREN DURING COVID-19 PANDIMIC.  Protocol Identification: 002SRC02082020 |  |
| --- | --- |
| **Patient and public involvement in research**  The researchers distributed the questionnaire online as the questionnaire will be distributed online on social media sites (WhatsApp- Facebook- Twitter) to be filled out personally. The questionnaire had a brief introduction explaining the nature of the research and confidentiality of the information that is given to participants. |  |
| **Study design**  Cross-sectional study was conducted during the period 1^st^ July 2020 to 30^th^ January 2021. | 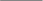 |
| **Setting and timeframe of research**  **Study design and duration: Cross-sectional study was conducted during the period 1^st^ July 2020 to 30^th^ January 2021.**  **Study area:** The study was conducted in different regions in Saudi Arabia |  |
| **Study groups**  Saudi parents living in Saudi Arabia during the COVID-19 outbreak. |  |
| **Subgroup analysis**  **(** KSA regions, gender, education, how many kids, ) | 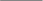 |
| **Recruitment** – **The Sampling Technique:** Random sampling technique was adopted to select the sample size.  **Data Collection Tool:**  A self-administered online disseminated questionnaire was used for data collection. It is composed of two main sections. Section 1 includes socio-demographic characteristics of the parents (child sex, mother education, and residency). The second section asks about physical activity, food, vaccination delay, supplements benefit, susceptibility, symptoms, protective precautions to children, and supplements. Permission to utilize the questionnaire was asked from the two main authors through email.  **Data Collection Technique:** The researchers distributed the questionnaire online as the questionnaire will be distributed online on social media sites (WhatsApp- Facebook- Twitter) to be filled out personally. The questionnaire had a brief introduction explaining the nature of the research and confidentiality of the information that is given to participants.  **Data Management and Analysis Plan:** All data were analyzed using SPSS 23 with using appropriate statistical methods for description and analysis. A P-value less than 0.05 was considered for statistical significance.  **Statistical analysis:** Data were entered and analyzed using Statistical Package for the Social Sciences (SPSS) version 17. Descriptive statistics were displayed as frequencies and percentages for categorical variables. Univariate analysis was performed to compare between each region, with the outcome, on the one hand, this was performed using Chi-squared test. |  |
| **Sample size** – The minimum sample size for this study has been decided according to Swinscow, as follows:  n = Z^2^ x P x Q  D^2^  Where:  n: minimum sample size  Z: The z-value for the selected level of confidence (1- α) = 1.96.  P: An estimated prevalence of having a positive attitude.  Q: (1 – 0.50) = 50%, i.e., 0.50  D: The maximum acceptable error = 0.05.  So, the calculated minimum sample size was:  n (minimum) = (1.96)^2^ X 0.50 X 0.50 = 384  (0.05) ^2^  532 participants were included in the study from 3 different geographical regions in the kingdom. |  |
| **METHODS - INTERVENTION AND CONSIDERATIONS** | |
| **Pre-intervention considerations** –N/A |  |

| **Intervention** –N/A |  |
| --- | --- |
| **Intra-intervention considerations** – NA | 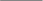 |
| **Operator details N/A** |  |
| **Quality control** –N/A | 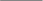 |
| **Post-intervention considerations** –N/A | 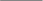 |
| **Statistics** –  Frequency Percent  **Parent gender**  Father 161 30.3  Mother 371 69.7  Total 532 100.0  **Mother education**  Didn't finish school 9 1.7  High school graduate 79 14.8  College 444 83.5  **Residency**  West 288 54.1  Eastern 112 21.1  Southern 11 2.1  Northern 4 .8  Central 117 22.0  **Children**  One 102 19.2  Two to three 189 35.5  more than three 241 45.3  **Physical Activity**  Increased 112 21.1  Decreased 197 37.0  No change 223 41.9  **Food**  always homemade food 237 44.5  Most of the time homemade food 275 51.7  Sometimes homemade food 18 3.4  Rarely homemade food 2 .4  **Vaccination delay**  Yes 74 13.9  No 114 21.4  No child to be vaccinated 344 64.7  **Supplements benefit**  Yes 343 64.5  No 42 7.9  Maybe 147 27.6  **Susceptibility**  Yes 243 45.7  No 289 54.3  **Symptoms**  Yes 360 67.7  No 172 32.3  **Protective precautions for children**  Hand wash 452 85.0  No kid goes for groceries 366 68.8  Face mask 423 79.5  No kid receives a delivery 327 61.5  Didn't have visitors 326 61.3  Going out for important stuff 371 69.7  **Supplements**.  Nothing 85 16.0  Honey 347 65.2  vitamins 208 39.1  Honey, Black seed, vitamins 184 34.6  Honey, Black seed 67 12.6  Honey, vitamins 55 10.4  Others 28 5.3 |  |
| **RESULTS** | |
| Our study included 532 participants, 69.7% of participated parents were mothers (females) and 30.3% were fathers (males). 83.5% of mothers were highly educated. Regarding residency, 54.1% of our sample were from the western region, 21.1% from the eastern region, 2.1% from the southern region, 0.8% from the northern region, and 22.0% from the central region.  The majority 41% didn’t observe any change in their children’s activity, the little less 37% agreed that it decreased their activity. The least said that children seemed more active. 44%-95% of the respondents ate from what they cooked ranging from all to most of the weekdays, less than 5% stuck with other sources. 64.5% of the parents believed that herbal meds and supplements boost their children's immunity. 27.6% were not sure. The rest 7.9% didn’t believe in their effect. 39% of the parents who had children in the age of vaccination, had to delay it, while 60 % stuck to the schedule. The severity of the symptoms, if it hits children comparing to the adults, was reported as 67.7% had milder symptoms and 32.3% thought no the presented with the same picture as adults (as showen in table 1). |  |
| **Intervention** –N/A |  |

|  |  |  |
| --- | --- | --- |
| 11a | **Outcomes** –N/A |  |
| 11b | **Tolerance** –N/A |  |
| 11c | **Complications** – N/A |  |
| **DISCUSSION** | | |
| 13 | **Discussion** – The huge numbers of cases and deaths COVID caused had all our attention, but simple things like diet and physical activity that can help with fighting the pandemic as a whole are left aside. A study done showed that the physical activity of people got reduced by 25% (6).  Among children and young people, there is compelling evidence suggesting that physical activity is important for health and well-being. Physical activity might improve not only cardiorespiratory and muscular fitness, cardiometabolic health, bone health, weight status, and cognition, but also reduce the risk of depression. The current physical activity recommendations suggest that children and young people aged 6-17 years should engage in 60 min/d or more of moderate-to-vigorous physical activity, of which vigorous physical activity should be included at least three days per week (7).  Diet and supplementation play a major role in human immunity and it has an association in modulating the immune responses COVID symptomatology (8). That also got affected people who had more frequent carbohydrate meals. mean weight was significantly higher during the curfew than before the curfew (9). On March 23, 2020, SPA -- An official source in the Ministry of Interior has stated that based on the order of the Custodian of the Two Holy Mosques King Salman bin Abdul-Aziz Al Saud to enforcing a curfew to limit the spread of the Novel Coronavirus. This lasted not less than two months (10). A study found that social distancing caused the case growth rate to reduce the total number of COVID-19 cases by approximately 1,600 reported cases at 7 days and the following days (5). Plus the variation that the chart showed along the timeline of the curfew of KSA (11), had us put people behavior and actions into consideration when dealing with outbreaks in general. And with that disturbing the rhythm of daily life routine of everyone, we wanted to know what change COVID made on the families that live in KSA, and what measure did the parents take to protect their children.  Starting from the physical activity of the children we asked the parents whether their children’s activity got affected. Regardless of the scientific accuracy of the measures taken by the parents during the pandemic, we asked them withier they believed in their effect and if they practiced any. And those were the supplements and food that were given to enhance the children’s immunity.  Moving to the precautions the Ministry of Health set to follow, we wanted to see how strict each parent was in keeping up with them, and we found that a good percentage of responses claimed that they followed most of the precautions. They let every member of the house including children wash their hands that got the highest percentage 85% in the other hand the responses with the people who didn’t have visitors at home or didn’t let any of the children meet outside visitors were the least 61%.  The primary immunization of children in KSA starts from birth till preschool age. It proved it is efficacy in eradicating some diseases and weakening others (12). Far from here, It has been reported that the number of MMR (measles, mumps, and rubella) vaccines delivered in England dropped by 20% during the first three weeks of the lockdown,8 and smaller falls were reported in infant vaccines in Scotland (13).  Children can be the secret host of COVID through witch is spreads fast without anyone knowing since they appear to present with less severe to no symptoms at all (14, 15).  Regardless of the absence of any study about the children's susceptibility to COVID, some parents believe that their children are more protected from COVID than the adults, and so we asked them if they believe in that. 45.7 % yes they answered children were more protected.  54% said no they were as susceptible as adults  The clinical presentation of covid-19 patients varies according to how each immune system will respond to it. The theory behind this is poorly understood although some research studies revealed major immunological parameter differences between individuals who tested positive (16), but the whole image is yet to be discovered. What we know so far are some theories based on many immunological studies that concluded that the age and the comorbidities of the patients are one of the main important players in the morbidity and mortality of the disease, but with no clear-cut lines (17). | 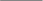 |
| 14 | **Strengths and limitations**  **Limitations** –the goal was to collect samples form KSA five regions with minimum size of 200 responses but the the regions that responded with more than 100 responses were only three , knowing that we exceeded the minimum sample size in total. |  |
| **CONCLUSION** | | |
| 15 | **Conclusions**  People sticking to precautionary measures were more relatable to what they believed not what they were forced to do, the increased number of cases at the beginning of the partial curfew proves that. Even though social gathering had its financial penalty the responses were greater with hand washing. Since they believed that their children are at less risk. Of dying or danger when getting COVID they did not mind those meeting people during that. Last but not least as for the vaccination being sabotaged by this even an organized health care system, isolated from the big scene should be built to establish a continuity of the primary care of children, minimizing the collateral damage in this disaster. |  |
| **DECLARATIONS** | | |
| 16a | **Conflicts of interest**  •N/A |  |
| 16b | **Funding**   - N/A |  |

|  |  |  |
| --- | --- | --- |
